# Electro-mechanically controlled assembly of reconfigurable 3D mesostructures and electronic devices based on dielectric elastomer platforms

**DOI:** 10.1093/nsr/nwz164

**Published:** 2019-11-04

**Authors:** Wenbo Pang, Xu Cheng, Haojie Zhao, Xiaogang Guo, Ziyao Ji, Guorui Li, Yiming Liang, Zhaoguo Xue, Honglie Song, Fan Zhang, Zheng Xu, Lei Sang, Wen Huang, Tiefeng Li, Yihui Zhang

**Affiliations:** 1 Applied Mechanics Laboratory, Department of Engineering Mechanics, Tsinghua University, Beijing 100084, China; 2 Center for Flexible Electronics Technology, Tsinghua University, Beijing 100084, China; 3 School of Microelectronics, Soft Membrane Electronic Technology Laboratory, Hefei University of Technology, Hefei 230601, China; 4 Zhejiang Lab, Hangzhou 311100, China; 5 State Key Laboratory for Manufacturing and Systems Engineering, School of Mechanical Engineering, Xi'an Jiaotong University, Xi'an 710049, China; 6 Center for X-Mechanics, Zhejiang University, Hangzhou 310027, China

**Keywords:** 3D assembly, buckling, reconfigurable structures, dielectric elastomers, reconfigurable RF circuits

## Abstract

The manufacture of 3D mesostructures is receiving rapidly increasing attention, because of the fundamental significance and practical applications across wide-ranging areas. The recently developed approach of buckling-guided assembly allows deterministic formation of complex 3D mesostructures in a broad set of functional materials, with feature sizes spanning nanoscale to centimeter-scale. Previous studies mostly exploited mechanically controlled assembly platforms using elastomer substrates, which limits the capabilities to achieve on-demand local assembly, and to reshape assembled mesostructures into distinct 3D configurations. This work introduces a set of design concepts and assembly strategies to utilize dielectric elastomer actuators as powerful platforms for the electro-mechanically controlled 3D assembly. Capabilities of sequential, local loading with desired strain distributions allow access to precisely tailored 3D mesostructures that can be reshaped into distinct geometries, as demonstrated by experimental and theoretical studies of ∼30 examples. A reconfigurable inductive–capacitive radio-frequency circuit consisting of morphable 3D capacitors serves as an application example.

## INTRODUCTION

The development of assembly techniques for 3D mesostructures is receiving rapidly increasing attention, due to the important implications across a broad range of areas, from microelectromechanical systems (MEMS) [1–4] and microelectronics [5–9] to biomedical devices [10–12] and metamaterials [13–15]. Fully compatible with well-established planar manufacturing technologies, the 3D assembly techniques are mostly applicable to a broad set of high-performance materials [16–24], including the most compelling electronic/optoelectronic materials, as well as a wide range of length scales (e.g. with feature sizes from several tens of nanometers to millimeters) [25–32]. Depending on the different deformation features, the existing 3D assembly techniques can be classified into four categories—rolling [3,33–36], folding [37–39], curving [40–47] and buckling [48–50] approaches [25]. As compared to the rolling, folding and curving approaches, the buckling-guided approaches offer access to a richer diversity of 3D geometries, since they incorporate strategic designs of 2D precursor structures and deformation substrates in combination with highly diversified loading schemes to achieve much more complex deformations during the assembly process [25,51–57]. In particular, the buckling-guided approaches usually rely on a pre-stretched elastomer substrate to serve as the assembly platform, where the micro-fabricated 2D precursor structures are integrated, with strong covalent bonding produced at selective locations. Release of the pre-stretch in this mechanical platform provides compressive forces and results in the 2D-to-3D transformation of the precursor structures, through coordinated translational/rotational motions and bending/twisting deformations. To achieve non-uniformly distributed 3D mesostructures, engineered elastomer substrates with either spatial variation of thickness/modulus or kirigami patterns were introduced as tailorable platforms that can offer desired strain distributions [58,59]. It is noteworthy that the existing studies based on buckling-guided approaches all exploit mechanical stages to apply the overall, pre-stretch deformations to the substrate, and the resulting 3D assembly follows in a global and concurrent manner at different spatial locations. A key limitation is the lack of ability to achieve on-demand local assembly with desired time sequences, and to reshape as-assembled mesostructures into distinct 3D configurations.

**Figure 1.**
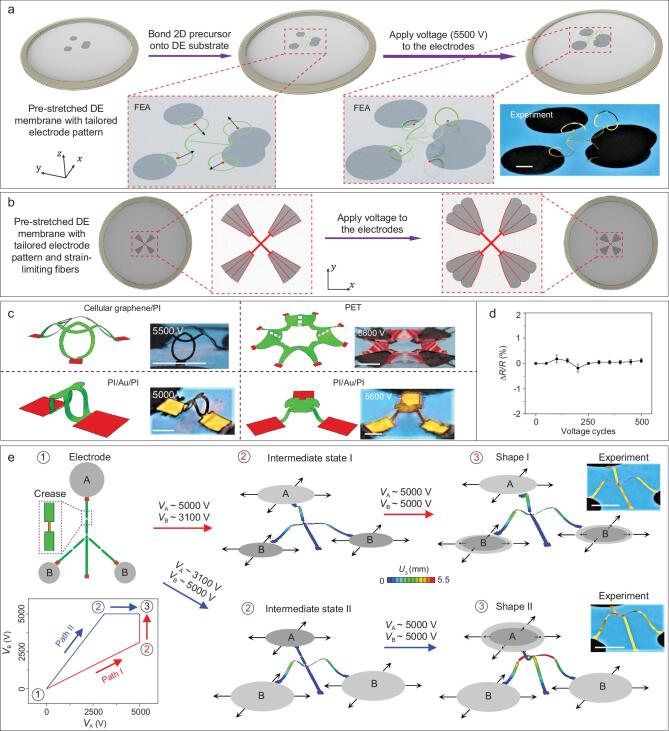
Electro-mechanically controlled deterministic assembly of 3D mesostructures in different material systems and length scales based on dielectric elastomer platforms. (a) Results of FEA that illustrate the process of electrically controlled 3D assembly based on dielectric elastomers (DE) substrates. Upon application of a high voltage to the compliant electrodes (dark gray), the 2D precursor structure (green) selectively bonded with the pre-stretched DE substrate is transformed into a deterministic 3D configuration. (b) Results of FEA that illustrate the deformation of composite DE substrates consisting of strain-limiting fibers (red lines), including the states before and after applying high voltages to the electrodes. (c) FEA and optical images of the assembled 3D mesostructures based on DE substrates, including structures in different material systems (cellular graphene/polyimide (PI) laminated film, polyethylene terephthalate (PET) film and PI/gold (Au)/PI laminated film) and length scales (ribbon width from 180 μm to 6 mm; ribbon thickness from 8 μm to 50 μm). (d) Electrical-resistance variation of the 3D structure made of cellular graphene/PI laminated film (top left in Fig. 1c) under cyclic electrical loading. (e) An example of a reconfigurable 3D mesostructure based on the electrically controlled DE substrate, with the use of two different loading paths. The 2D precursor structure, 3D FEA results and optical images are shown following the two different paths (I and II). Colors in FEA results represent the magnitude of the out-of-plane displacement of 3D mesostructures. Scale bars: 8 mm in (a); 8 mm for the top two images in (c); 0.8 mm for the bottom two images in (c) and 9 mm in (e).

Here, we present a set of strategies to overcome this limitation, where highly deformable dielectric elastomers (DE) capable of sequential, local actuation serve as a platform for buckling-guided 3D assembly. Dielectric elastomers have been widely used as a type of high-performance soft actuators for applications in soft robotics, biomedical devices, optical devices and many other areas [60–64]. This work introduces strategic designs of electrode layouts and strain-limiting fibers [65] in DE substrates, which allows the utility of electrical actuation and electro-mechanically coupled deformations to achieve complex strain distributions, with capabilities of sequential, local loading and strain isolation. As validated by experimental measurements, the theoretical modeling of electro-mechanically coupled deformations, implemented with simulations of finite element analyses (FEA), can predict accurately the distributions of strain components in DE substrates. These capabilities allow access to precisely tailored 3D mesostructures during electro-mechanically controlled assembly, whose geometries can be reshaped rapidly (∼1 s) through electrical actuation. Demonstrations encompass experimental and theoretical studies of nearly 30 examples in different length scales (from micrometer to millimeter scales) and material types (e.g. metals, polymers, and cellular graphene), including structures that resemble frogs, eyeglasses, starfishes and domes and those that can be reshaped between two distinct stable geometries. A reconfigurable inductive–capacitive (LC) radio-frequency (RF) circuit enabled by an electrically controlled 3D capacitor serves as a device demonstration, which suggests prospects of applications in wireless communication.

## RESULTS AND DISCUSSION

### Conceptual illustration of 3D assembly with DE substrates

Figure 1a provides results of finite element analyses (FEA; see ‘Methods’ section for details) that schematically illustrate the assembly process of 3D mesostructures based on electrically controlled DE substrates. A free-standing DE membrane (VHB, 3M, thickness 1 mm) is pre-stretched (e.g. pre-strain of ∼250%) to offer an enhanced actuation performance (see Supplementary 1a for the detailed manufacturing process) [66]. Then pre-designed 2D precursors (see ‘Methods’ section for details of the fabrication) are transferred onto the pre-stretched DE membrane, with strong bondings (four free ends of the ribbons in Supplementary Fig. 1a marked by red, available as Supplementary Data at NSR online) selectively distributed at the interface. Here, shadow masks with the patterns of the bonding sites allow accurate positioning of 2D precursors during the transfer printing process. Anti-stick powders (talcum powder) are exploited to reduce the adhesion energy at the non-bonded regions, avoiding the failure of 3D assembly due to the strong adhesion of the DE membrane (VHB 4910, 3M). It should be pointed out that the anti-stick powders may be unnecessary if the DE membrane (VHB 4910, 3M) is replaced by other dielectric elastomers (e.g. silicon rubber—Nusil, CF19–2186) with much lower adhesion energy. The applied voltages on the compliant electrodes (MG Chemicals, USA) at the top/bottom surfaces induce Maxwell forces that expand the dielectric membrane biaxially along the in-plane direction. Such actuation deformations result in inward movements of four bonding sites and trigger the compressive buckling of 2D precursors, transforming them into 3D configurations, through coordinated spatial bending/twisting deformations as well as translational/rotational motions.

Strain-limiting fibers can be embedded in the DE substrates to achieve non-equal biaxial in-plane expansion during the electrical actuation, which can enhance the complexity of strain distribution and enrich the diversity of accessible geometries during 3D assembly. Fibers with well-designed structural patterns and thicknesses are able to limit not only the deformation along specified linear directions, but also the deformation along desired radial directions. These fibers are typically placed on the bottom surface of DE platforms, and thereby do not affect the transfer printing process. Figure 1b presents a schematic illustration of the deformation of the composite DE substrates with strain-limiting fibers fabricated by 3D printing method, induced by the application of voltage (see Supplementary Fig. 1b for manufacturing process and ‘Methods’ section for preparation of fibers). Here, the 3D printing method is adopted for the rapid prototyping of strain-limiting fibers with diverse geometries.

Figure 1c provides results of finite element analyses (FEA; see ‘Methods’ section for details) and experiments for the assembly of 3D structures in a range of different materials (cellular graphene/polyimide (PI) laminated film [21]; polyethylene terephthalate (PET) film; PI/gold (Au)/PI laminated film; see ‘Methods’ section for details) and length scales (ribbon width from 180 μm to 6 mm; ribbon thickness from 8 μm to 50 μm). The designs of electrode layouts and 2D precursors are provided in Supplementary Fig. 2. Here, FEA can predict accurately the electro-mechanical deformations in the pre-stretched DE substrate under a prescribed voltage, and the resulting post-buckling process of 2D precursor structures. This is evidenced by the good agreements of predicted 3D configurations with optical images for all of the four examples in Fig. 1c. The assembled 3D mesostructures are mechanically robust, with capabilities to endure cyclic electrical loading and unloading. Figure 1d shows the electrical resistance of the cellular graphene/PI mesostructure (in the top-left panel of Fig. 1c) wired from two of the bonding regions, during cyclic electrical loading with an amplitude of 5500 V and a frequency of ∼0.5 Hz. The relative variation of the electrical resistance is below 0.17% for 500 cycles.

Figure 1e, Supplementary Fig. 3 and Supplementary Movie 1 illustrate the utility of electrically controlled, local, sequential loading enabled by the DE platform to achieve a morphable 3D mesostructure whose shape can be stabilized between two distinct geometries [51]. In this example, the 2D precursor consists of three identical ribbons, each with two creases (with a reduced width of 200 μm, as compared to 800 μm in the other regions), and a supporting ribbon at the bottom of the design, as shown in the top-left panel. Two types of electrodes (A and B) are designed to offer sequential actuation in the DE platform. Specifically, two different loading paths are exploited, denoted as (Path I: [*V*_A_ = *V*_B_ = 0] → [*V*_A_ = 5000 V, *V*_B_ = 3100 V] → [*V*_A_ = 5000 V, *V*_B_ = 5000 V]) and (Path II: [*V*_A_ = *V*_B_ = 0] → [*V*_A_ = 3100 V, *V*_B_ = 5000 V] → [*V*_A_ = 5000 V, *V*_B_ = 5000 V]) (bottom-left panel in Fig. 1e). During ‘Path I’ loading, the supporting ribbon remains almost flat and attached to the substrate during the entire process, resulting in the formation of a 3D structure (Shape I) with three arches connected at the center. In contrast, ‘Path II’ loading yields a different configuration (Shape II) where the center is elevated and the supporting ribbon is evidently curved. Both shapes (I and II) are stable and can be transformed reversibly into each other, by removing and applying the electrical loading with different sequences.

**Figure 2.**
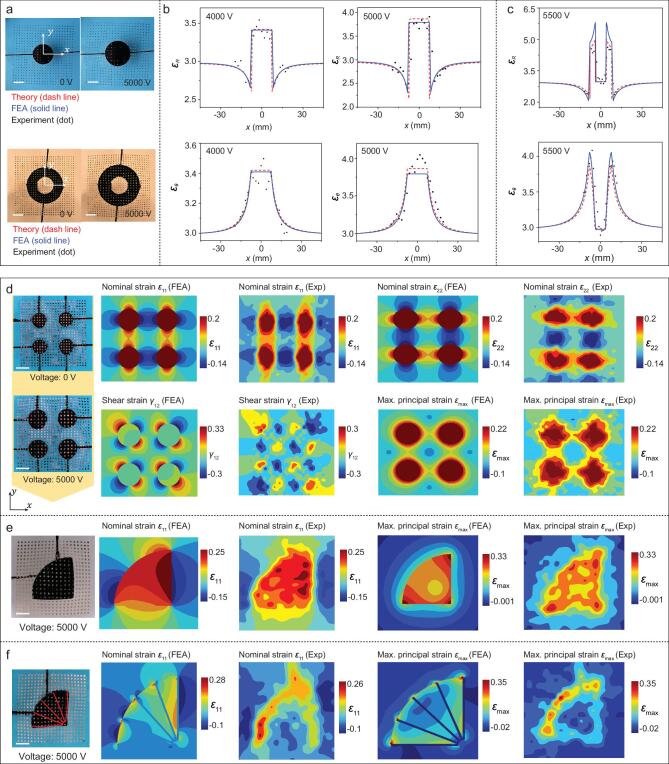
Theoretical and experimental studies on the distributions of strain components in DE substrates with four representative electrode layouts. (a) Experimental images of circular and annular electrodes with and without the voltage application (i.e. 0/5000 V). (b) Theoretical, FEA and experimental results of the distributions of circumferential and radial normal strain (${\varepsilon}_{\theta}$ and ${\varepsilon}_{\mathrm{R}}$, respectively) along the *x* direction of the circular electrode, at two different levels of applied voltages. (c) Similar results for the annular electrode at an applied voltage of 5500 V. (d) Optical images of the unactuated (0 V) and actuated (5000 V) configurations of the DE substrate with 2 $\times$ 2 array of circular electrodes and the distributions of strain components (normal strain ${\varepsilon}_{11}$ and ${\varepsilon}_{22}$, shear strain ${\gamma}_{12}$ and maximum principal strain ${\varepsilon}_{\mathrm{max}}$) determined from the experiment and FEA. Arrays of displacement markers are used for strain visualization. Colors in the contour plots denote the magnitude of strain components. (e) Optical images of the actuated (5000 V) configurations of the DE substrate with a sector electrode and the distributions of strain components (normal strain ${\varepsilon}_{11}$ and maximum principal strain ${\varepsilon}_{\mathrm{max}}$) determined from the experiment and FEA. (Details of ${\varepsilon}_{22}$ and ${\gamma}_{12}$ can be found in Supplementary Fig. 6b). (f) Similar results for the case of the sector electrode with strain-limiting fibers. Details of ${\varepsilon}_{22}$ can be found in Supplementary Fig. 6c. The deformed strain-limiting fibers are marked by red in the optical image. Scale bars: 5 mm.

### Experimental and theoretical studies of electro-mechanically coupled deformations in DE substrates

To offer versatile loading combinations and sequences for 3D assembly, the pattern of electrode layouts can be tailored to achieve a variety of desired strain distributions in DE substrates, with FEA as a design platform. Note that several different approaches were developed to predict the non-linear electro-mechanical deformations of the DE platform under electrical/mechanical loadings [67–69]. In the current study, the FEA were carried out using commercial software (ABAQUS), in which equivalent in-plane forces perpendicular to the edge of regions covered by electrodes were applied to account for the effect of Maxwell stress [66] (see ‘Methods’ section for details). Quantitative measurements by tracking the displacements of deposited metal external arrays allow us to obtain the in-plane strain fields of DE substrates experimentally (see ‘Methods’ section for details). Figure 2a–c and Supplementary Fig. 4 show that the distributions of circumferential and radial nominal strain (${\varepsilon}_{\theta}$ and ${\varepsilon}_{\mathrm{R}}$ respectively) calculated based on the FEA agree quantitatively with both experimental measurements and theoretical results based on Suo’s theory [66,70] (see Supplementary Note, available as Supplementary Data at NSR online), for the cases of circular and annular electrodes, even at relatively high voltages (e.g. 5500 V). It is noteworthy that both the electrically induced circumferential and radial nominal strain are almost zero within the inner circle of the annular electrode (Fig. 2c and Supplementary Fig. 4b), as evidenced by the magnitude of electrically induced nominal strain that is less than 0.8% at 5500 V according to FEA.

Figure 2d–f and Supplementary Figs 5 and 6 provide combined experimental and numerical results of in-plane strain distribution for four representative electrode layouts, including annular electrode, circular electrode, sector electrode without any fibers, and sector electrode with strain-limiting fibers. For a 2 $\times$ 2 array of circular electrodes (Fig. 2d), the distributions of three strain components (${\varepsilon}_{11}$, ${\varepsilon}_{22}$ and ${\gamma}_{12}$) as well as the in-plane maximum principal strain (${\varepsilon}_{\mathrm{max}}$) predicted by FEA all agree well with the experimental results. Here, ${\varepsilon}_{11}$and ${\varepsilon}_{22}$ are the two in-plane nominal normal strain components, and ${\gamma}_{12}$ represents the in-plane engineering shear strain. These results show very slight mechanical interactions of the neighboring electrodes, indicating an excellent strain isolation, in the condition that the spacing is equal to (or larger than) the electrode diameter before the electrical loading. The normal strain components (${\varepsilon}_{\mathrm{x}}$) are alternatively negative and positive across the line connecting the centers of the two electrodes (Supplementary Fig. 7). Figure 2e and f, Supplementary Figs. 6b and c, and Supplementary Fig. 8 elucidate the strain-limiting effect of fibers. Due to the relatively large stiffness, the fibers along the radial direction undergo negligible deformations, thereby effectively restricting the radial deformations of the DE platform. In all of those cases, the difference between FEA and experiments can be mainly attributed to the limited number of metal dots that poses constraints on the spatial resolution of strain distributions.

### Assembly of 3D structures through the DE platform

**Figure 3.**
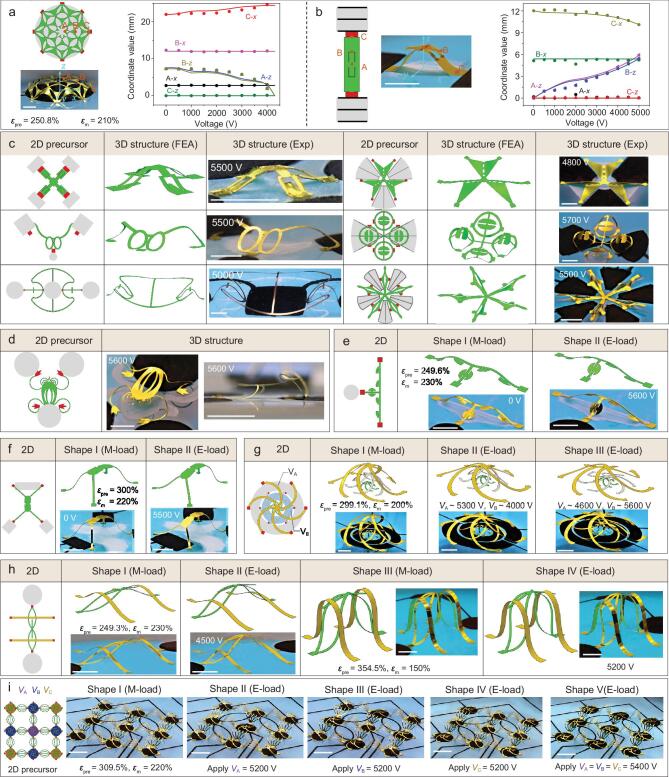
Experimental and FEA results of diverse reconfigurable 3D structures based on electro-mechanically controlled assembly. (a, b) Quantitative comparisons of FEA and experimental results for the dome-like mesostructure actuated by a circular electrode (a) and the ribbon mesostructure actuated by two rectangular electrodes with strain-limiting fibers (b). Three mark points (A, B and C) are selected to quantitatively characterize the electrically actuated deformation of 3D mesostructures at different voltages, where A-*x* (or A-*y*, A-*z*) represents the *x* (or *y*, *z*) coordinate value of point A. Dots and lines denote experimental results and FEA predictions, respectively. (c) Experimental and FEA results of six representative mesostructures achieved by electrically controlled 3D assembly. The electrodes, fibers and bonding sites are marked by gray, black and red in the schematics of the 2D precursor, made of Cu/PET (1 μm/50 μm) for the bottom-left mesostructures, and Al/PET (2.5 μm/30 μm) for the others. (d) Optical images of the frog-like mesostructure and its 2D precursor (details of the FEA results can be found in Supplementary Fig. 11). (e–h) Experimental and FEA results of four representative reconfigurable mesostructures with coupled mechanical and electrical loadings. ${\varepsilon}_{\mathrm{pre}}$ and ${\varepsilon}_{\mathrm{m}}$ denote the pre-stretching strain initially applied to the DE substrate and the tensile strain after the mechanical loading process, both with respect to the undeformed configuration of the DE substrate. ‘M-load’ and ‘E-load’ represent ‘Mechanical loading’ and ‘Electrical loading’, respectively. (i) A 3D reconfigurable network mesostructure consisting of two different ribbon elements, demonstrating the excellent local strain control through the coupled electrical/mechanical loadings of the DE substrates. Three types of electrodes (A, B and C) are marked by purple, dark blue and deep yellow, respectively, in the leftmost panel. Scale bars: 10 mm in (a, b), (d, e) and (g–i); 6 mm in (c); 8 mm in (f).

Accurate modeling of the electro-mechanically coupled deformations in DE substrates, together with precise post-buckling analyses, allows rational designs of electrode layouts, 2D precursor patterns, bonding sites and loading magnitude to yield target 3D configurations. Figure 3a, b and Supplementary Fig. 9 present two representative examples, in which the geometries of 3D mesostructures can be tuned through electrical actuation, in a quantitative manner. The mesostructure in Fig. 3a was mechanically assembled first by releasing the pre-strain from ${\varepsilon}_{\mathrm{pre}}$ = 250.8% to an intermediate value (${\varepsilon}_{\mathrm{m}}$ = 210%), where both ${\varepsilon}_{\mathrm{pre}}$ and ${\varepsilon}_{\mathrm{m}}$ are defined with respect to the undeformed configuration of the DE substrate. Application of the voltage then expands the distance between neighboring bonding sites, thereby lowering the height of the architecture, until it reaches the flat (2D) configuration at 4300 V. Three mark points (A, B, C) are selected to quantitatively characterize the electrically actuated deformation of 3D structures at different voltages. The variations of their coordinate components show good agreements between FEA and experiments, as shown in the right panel of Fig. 3a. Furthermore, the mesostructure in Fig. 3a can be reshaped rapidly (less than 1 s) once the voltage is applied compared with the mechanical loadings adopted in previous studies (larger than 30 s) (see Supplementary Movie 2). Here the speed of loading is limited by the relatively high viscosity of the VHB membrane. To further improve the actuation speed and the actuation efficiency, other dielectric elastomers with lower viscosities can be exploited. The kirigami arch mesostructure in Fig. 3b shows a 2D configuration at the zero-voltage state. In this case, the strain-limiting electrode induces a uniaxial compression to the 2D precursor structure upon application of the voltage, and therefore the amplitude of the kirigami arch increases gradually with increasing voltage. The coordinates of the three mark points (A, B, C) in Fig. 3b predicted by FEA also match the experimental results well.

Figure 3c presents six demonstrative examples of electrically controlled 3D assembly, including 2D precursors (with electrodes and bonding sites marked by gray and red, respectively), FEA predictions and experimental images of final 3D mesostructures. The mesostructures in the left column are assembled through the lateral buckling controlled by the voltage applied to the electrodes that do not contain any strain-limiting fibers. The second example resembles an eyeglass, which is formed by applying a voltage of 5500 V equally to the three electrodes. The assembly of the third mesostructure involves a coordinated process of tensile buckling and compressive buckling, where the curvy ribbon structure connected to the two central bonding sites undergoes tensile buckling, and the two outer ribbons experience compressive buckling. The designs in the right column are achieved by exploiting strategically designed fibers in the electrodes, and the resulting 3D geometries are unachievable using conventional elastomer substrates. Here, the fibers mainly restrict the radial expansion, such that the compressive buckling along the circumferential direction can be realized to form 3D mesostructures with a certain degree of rotational symmetry. In particular, the last mesostructure with a five-fold rotational symmetry resembles a starfish. Four additional examples of electrically controlled 3D assembly are provided in Supplementary Fig. 10. Figure 3d and Supplementary Fig. 11 show a frog-like mesostructure enabled by concurrent tensile buckling and compressive buckling during the electrically controlled 3D assembly, such that the gestures of forelegs and hindlegs can resemble those of real frogs. In the aforementioned examples, the largest electrically induced strain (nominal linear strain) of the DE platform is ∼80%, which is smaller than the mechanical strain (nominal linear strain, typically larger than 100%) achievable with the traditional silicone substrate.

Based on the DE platform, coupled electrical and mechanical loadings can be achieved easily to enrich the capability of geometrical control during the 3D assembly. For example, an equal biaxial compression followed by electrical loading can be exploited to reshape the mechanically assembled 3D mesostructures into a distinct configuration, as shown in Fig. 3e–i and Supplementary Fig. 12a. Specifically, the mesostructure in Fig. 3f and Supplementary Movie 3 resembles an insect, and exhibits a different gesture after rapid electrical actuation (∼1 s). Figure 3g shows that a three-layer cage structure can be formed through biaxial compression during the release of pre-strain in the DE substrate. Such a three-layer cage structure can be reshaped through two individually addressable electrodes, such that the middle layer can selectively be in contact with the top layer (shape II) or the bottom layer (shape III). This reconfigurable characteristic can be potentially utilized to design reconfigurable electronic components (e.g. inductors and antennas). The mesostructure in Fig. 3h elucidates a design where the state of contact between the suspended straight ribbons at the bottom layer (in green) and arch-shaped ribbons at the top layer (in yellow) can be tuned by adopting different electro-mechanical loadings. Reversible local actuation of the 3D mesostructure is also possible by adopting designs of electrode arrays with the feature of strain isolation, which is otherwise unobtainable using the mechanically guided assembly approaches reported previously. Figure 3i and Supplementary Fig. 12b present a periodical array of a 3D ribbon network assembled through biaxial compression during the release of pre-strain in the DE substrate. Appropriate circuit designs allow the classification of nine circular electrodes into three groups (marked by different colors in the leftmost panel), such that the electrodes in each group can be addressed individually. When the voltage (∼5200 V) is applied only to the Group-A electrode in the center, the central mesostructure is flattened, while the other 3D mesostructures are almost unaffected, especially those sitting on the other electrodes. Similarly, the voltage (∼5200 V) applied only to four Group-B (or Group-C) electrodes results in the flattening of four associated mesostructures, with negligible influences on the mesostructures sitting on the other electrodes.

**Figure 4.**
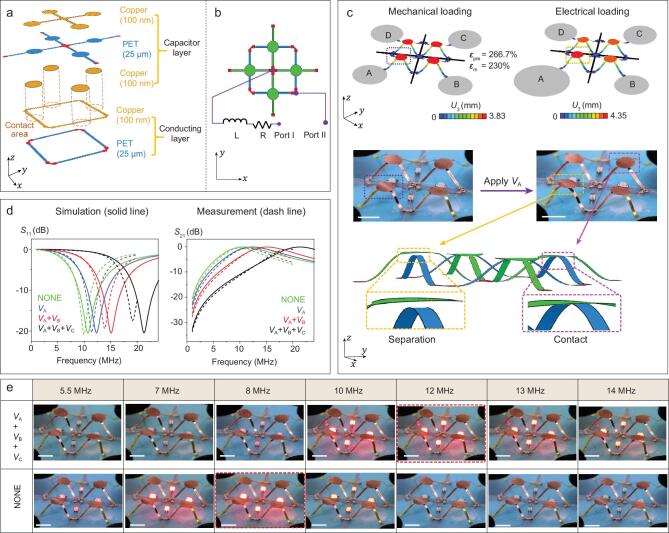
A reconfigurable inductive–capacitive (LC) radio-frequency (RF) circuit consisting of four morphable 3D capacitors that can be addressed individually. (a) Exploded view of the 2D precursor structures, consisting of separable bi-layers (capacitor layer on the top and conducting layer on the bottom). (b) A circuit diagram of the functional device in the planar state. The inductance and resistance components are externally connected. (Details of the equivalent circuit can be found in Supplementary Fig. 13a). (c) Optical images and FEA results showing the working principle of the device. Colors in FEA represent the magnitude of the out-of-plane displacement of the 3D mesostructure. Gray areas and the black crosses represent four different compliant electrodes (A, B, C and D) and strain-limiting fibers, respectively. The 3D mesostructure in the left column is obtained by equal biaxial mechanical loadings. The detailed fabrication process is shown in Supplementary Fig. 14b. After applying a voltage of *V*_A_ = 5200 V to electrode A, the adjacent 3D mesostructure can be transformed into a new configuration (from left to right), in which the top and bottom layers are separated from each other. (d) Measured (dash line) and simulated (solid line) *S*_11_ and *S*_21_ versus frequency for four states. ‘NONE’, ‘*V*_A_’, ‘*V*_A_ + *V*_B_’ and ‘*V*_A_ + *V*_B_ + *V*_C_’ denote the cases of ‘no applied voltage’, ‘*V*_A_ = 5200 V’, ‘*V*_A_ = *V*_B_ = 5200 V’ and ‘*V*_A_ = *V*_B_ = *V*_C_ = 5200 V’, respectively. (e) Demonstration of the tunable capacitor device to adjust the light intensity of LEDs. Four commercial LEDs are connected in parallel with the morphable 3D capacitors. Two representative states of the LC-RF circuit are compared herein, in which all of the four capacitors are switched on or a single capacitor is on, thereby shifting the resonant frequency of the circuit considerably (a detailed equivalent circuit can be found in Supplementary Fig. 13b). Scale bars: 6 mm.

### A reconfigurable inductive–capacitive radio-frequency (LC-RF) circuit enabled by a morphable 3D capacitor

Versatile design freedom to access to diverse 3D geometries and powerful capabilities to reshape assembled mesostructures into distinct 3D configurations pave the way for unusual electronics with functionally reconfigurable features. Figure 4 demonstrates an example of a morphable 3D capacitor that enables a reconfigurable LC-RF circuit capable of shifting the resonant frequency on demand. Figure 4a illustrates schematically the separable bi-layer construction of the 2D precursor, where the upper layer, called the ‘capacitor layer’, has thin copper layers (100 nm) selectively deposited on the top and bottom surfaces of the PET layer (25 μm) to form parallel-plate capacitors, and the lower layer, called the ‘conducting layer’, has a single copper layer fully deposited on top of the PET layer (25 μm). Partial release of the pre-strain (${\varepsilon}_{\mathrm{pre}}$ = 266.7%, ${\varepsilon}_{\mathrm{m}}$ = 230%) in the DE substrate ensures a good contact between the lower and upper layers in the mechanically assembled device, and thereby all of the four capacitors are electrically conducting in the circuit (Fig. 4b and Supplementary Fig. 13a). We introduced a cross-shaped fiber on top of the DE substrate after the mechanical assembly, such that excellent strain isolation can be realized during the electrical actuation (Supplementary Fig. 14a). As a result, when the voltage is applied only to electrode A, the lower and upper layers are separated only in the adjacent capacitor while the other three capacitors remain electrically connected in the circuit (Fig. 4c). Similarly, the application of voltage to two (or three) electrodes leads to the formation of two capacitors connected in parallel (or a single capacitor) in the circuit. Thereby, the capacitance of the device can be reconfigured rapidly (within ∼1 s) through application of voltage to a different number of electrodes.

When the above morphable capacitor is connected with inductive and resistive components in an RF circuit (see Fig. 4b and Supplementary Fig. 13a for the equivalent circuit), the resonant frequency can be reconfigured on demand. Figure 4d shows the measured and simulated *S*_11_/*S*_21_ versus the frequency for the four different states, corresponding to the application of voltage to 0/1/2/3 electrodes, in which *S*_11_ and *S*_21_ represent the reflection coefficient and the insertion loss of the input signal, respectively. According to the theory of LC resonant circuits, the resonance frequency (*f*) can be given by $f=1/2 \pi \sqrt{LC}$, where *L* is the inductance and *C* is the capacitance. As the number of parallel capacitors in action increases, the capacitance value increases, leading to a shift of the resonance frequency to the lower band. Figure 4d demonstrates that the electrical actuation is able to substantially and continually achieve reconfigured resonant frequencies from ∼10.7 MHz to ∼21.3 MHz, while maintaining the reflection coefficient and the insertion loss relatively stable at −20.5 dB and − 0.1 dB, respectively. The results of electromagnetic simulations (see ‘Methods’ section for details) match the measured data very well.

Figure 4e provides a further experiment to demonstrate the frequency reconfigurability, in which four identical LEDs are connected in parallel at two ends of the capacitors (see Supplementary Fig. 13b, Supplementary Movie 4 and ‘Methods’ section for details). Considering that the dielectric loss and radiation loss of the demonstrative circuit is negligible, when the reflection coefficient achieves the minimum value at the resonant frequency, the circuit load (LEDs) should absorb the largest power from the source, suggesting that the four LEDs achieve the maximum brightness. A comparative experiment was carried out, in which the voltages were applied to three or none of the four electrodes. In these two conditions, the LED brightness reaches a peak clearly at distinct frequencies (∼12 MHz versus 8 MHz). It is noteworthy that when the capacitive network in Fig. 4a is loaded with LEDs (shown in Fig. 4e), parasitic effects such as additional inductive or capacitive parameters associated with LEDs lead to the offset of the resonant frequency from that of the original capacitive network. Furthermore, the uncontrollable electrical performance tolerance of the capacitors also contributes to the discrepancy between the desired and the measured frequency for achieving the maximum brightness. The design concepts introduced herein can be extended to the GHz band, and possible future applications include multi-channel 5G or post-5G wireless communications, and miniaturization of microwave systems.

## CONCLUSION

In summary, the design concepts, assembly strategies and validated modeling methods presented here establish the DE substrate as a powerful platform for the electro-mechanically controlled assembly of 3D mesostructures in a range of advanced materials, with the feature sizes spanning microscale to centimeter-scale. Combined theoretical and experimental studies show that strategic designs of electrode layouts and strain-limiting fibers in DE substrates can enable sequential, local and fast loading with desired strain distributions (with a maximum nominal linear strain of ∼80%), which allows access to precisely tailored 3D mesostructures and reconfigurable mesostructures. A morphable capacitor is designed and utilized to demonstrate a wide band frequency reconfigurable LC-RF circuit, holding promise for applications in wireless communication. The electro-mechanically controlled assembly approach developed herein can also be potentially used in the areas of optical metamaterials, flexible electronics, and MEMS/NEMS.

## METHODS

### Fabrication of 2D precursors

The 2D precursors made of PI/Au/PI films were fabricated by spin-coating (3000 rpm for 30 s) and curing (180°C for 12 min) a layer of poly(methyl methacrylate) (PMMA, Microchem, USA) (∼100 nm) onto a silicon wafer, followed by spin-coating (5000 rpm for 45 s) a layer of polyimide (PI, Sigma-Aldrich, USA) (∼8 μm) and curing (180°C for 2 h) on the PMMA layer. Depositing thin layers of metal (∼10 nm Cr/∼200 nm Au) by electron beam evaporation, and spin-casting another layer of PI (∼8 μm) finishes the preparation of the PI/Au/PI tri-layer film. Femtosecond laser cutting (Rofin, Germany) then defines the desired patterns of the 2D precursors. Immersing the silicon wafer in acetone for 10 min dissolves the PMMA layer, allowing the retrieval of the 2D precursors from the wafer.

The 2D precursors made of cellular graphene/PI films were prepared by ablating the PI film with a CO_2_ laser cutting machine (VLS2.30, USA) in scanning mode, generating cellular graphene on the top surface of the PI films [21].

The 2D precursors made of PET films, Cu/PET films or Al/PET films were prepared by automatic mechanical cutting (Silhouette CAMEO, USA) or laser cutting (VLS2.30, USA) of commercial thin films/foils (∼50 μm PET, ∼1 μm Cu/∼50 μm PET or ∼2.5 μm Al/∼30 μm PET) into the desired 2D shapes.

### Fabrication of strain-limiting fibers

The strain-limiting fibers were fabricated by 3D printing (Stratasys Objet Eden260VS 3D printer, USA) with an elastic modulus of ∼2 GPa (VeroBlue RGD840).

### Experimental visualization of strain fields of DE substrates

Strain visualization of the DE platform with tailored electrode patterns began with the sputtering of an array of copper dots (∼5 nm Ti/∼100 nm Cu, diameter = 400 μm, and pitch = 1 mm) on the surface of pre-stretched DE substrates with the aid of shadow masks. A series of optical images with different applied voltages were captured by a digital camera. The coordinate positions of the dots were processed automatically by image processing software (PhotoModeler) and the strain distributions could then be computed by MATLAB (version 2017b).

### Fabrication of 3D tunable capacitors for the LC-RF devices

The 2D precursors of the tunable capacitor device were made of composite films (∼30 μm PET/∼100 nm Cu for the ‘conducting layer’ and ∼100 nm Cu/∼30 μm PET/∼100 nm Cu for the ‘capacitor layer’). The fabrication of 3D LC-RF devices began with transferring the 2D precursors of the ‘conducting layer’ and ‘capacitor layer’ onto the DE platform, followed by selective bonding of the 2D precursors onto the DE platform. Releasing the pre-strain of DE platforms uniformly (mechanical loading process) allows transformation of 2D precursors into 3D mesostructures. Attaching the cross-shaped strain-limiting fibers onto the bottom surface of the DE platform enables tight bonding, thanks to the high viscosity of the DE membrane (VHB 4910, 3M, USA). Application of voltages to the electrodes reshapes the 3D mesostructures into new configurations (electrical loading process).

In the experiments to adjust the light intensity of LEDs, the parameters of the exploited components include: the commercial LEDs (red light, rated voltage = 1.5 volts, rated current = 5 ∼ 20 mA and rated power = 0.01 W); the external inductor (1.5 μH); the external resistance (1 $\Omega$) and the signal generator (sinusoidal alternating current (AC) signal and peak-to-peak voltage = 1.5 volts, Tektronix AFG 2021, USA).

### Electromagnetic measurements

Two port S-parameters were measured using a network analyzer (Agilent E5071B, USA). The capacitance value of each 3D capacitor is approximately 36.7 pF according to the measurement of the capacitance meter. The measured four resonant frequencies in Fig. 4d are 9.93 MHz, 11.45 MHz, 13.92 MHz and 19.15 MHz, respectively.

### Computational models of DE platforms

The commercial FEA software (ABAQUS) was exploited to predict the electrical deformations of the DE platforms. According to a previous study [66], the 3D Maxwell stress applied to the DE membrane can be modeled approximately using an equivalent plane stress. Here, four-node shell elements (S4R) were applied to model the DE membrane, and the Arruda–Boyce hyperelastic constitutive model was exploited to simulate the large deformations of the DE substrates. The ‘shell edge load’ (ABAQUS) was applied to the edges of the electrode regions, and the magnitude was calibrated by a set of experiments in the case of a circular electrode at different applied voltages. Convergence of mesh sizes was tested to ensure computational accuracy.

### Computational models of electromagnetic simulations

The electromagnetic finite element method was exploited to model the electrical performance of the tunable capacitor. The fabricated reconfigurable capacitor consists of two slightly bent parallel metal plates, in which the PI layer serves as a dielectric interlayer. For simplicity, the curvy effect of metal plates on the capacitance can be ignored in the targeted low-frequency band. Taking the low impact of the ohmic and dielectric loss into consideration, a cylindrical structure with a perfect electric conductor (PEC) boundary condition setting on the two sides as metal plates was modeled in the high-frequency structure simulator (HFSS). The relative permittivity (${\varepsilon}_{\mathrm{r}}$ = 4.6) of the inter-dielectric material was derived from measured samples.

## Supplementary Material

nwz164_Supplemental_Files
