# Supplementary material for: Electro-mechanically controlled assembly of reconfigurable 3D mesostructures and electronic devices based on dielectric elastomer platforms
Source: Natl Sci Rev. 2019 Nov 4;7(2):342–54. doi: 10.1093/nsr/nwz164 (PMC8288899; doi:10.1093/nsr/nwz164)
Supplement: nwz164_Supplemental_Files [file nwz164_supplemental_files.zip › Supporting information.pdf]

Supplementary information for
Electro-mechanically controlled assembly of reconfigurable 3D mesostructures and electronic devices based on dielectric elastomer platforms

Wenbo Pang[1,2], Xu Cheng[1,2], Haojie Zhao[3], Xiaogang Guo[1,2], Ziyao Ji[1,2], Guorui Li[4], Yiming Liang[4], Zhaoguo Xue[1,2], Honglie Song[1,2], Fan Zhang[1,2], Zheng Xu[1,2,5], Lei Sang[3], Wen Huang[3], Tiefeng Li[6], Yihui Zhang[1,2,*]

[1]*AML, Department of Engineering Mechanics, Tsinghua University, Beijing 100084, China;*

[2]*Center for Flexible Electronics Technology, Tsinghua University, Beijing 100084, China;*

[3]*School of Microelectronics, Soft Membrane Electronic Technology Laboratory, Hefei University of Technology, Hefei 230601, China;*

[4]*Zhejiang Lab, Hangzhou 311100, China.*

[5]*State Key Laboratory for Manufacturing and Systems Engineering, School of Mechanical Engineering, Xi'an Jiaotong University, Xi'an 710049, China;*

[6]*Center for X-Mechanics, Zhejiang University, Hangzhou 310027, China;*

* Corresponding author. Email: yihuizhang@tsinghua.edu.cn

Supplementary Note

1. A theoretical model of electro-mechanical deformations in the dielectric elastomer (DE) substrates with annular electrodes

For the DE substrates with annular electrodes, a theoretical model can be established based on the finite strain theory and Suo's model [1,2]. The theoretical model involves three deformation states of the DE substrates (see **Supplementary Fig. 4c**, available as Supplementary Data at NSR online). The 'freestanding state' (i.e., the reference configuration) corresponds to the DE membrane without any pre-stretch. The 'voltage off state' corresponds to the pre-stretched DE membrane with no applied voltages, and the 'voltage on state' corresponds to the pre-stretched DE membrane with applied voltages. Based on Suo's model [1], the effect of the voltages on the DE membranes is equivalent to an equal-biaxial planar Maxwell stress of εE^2. Specifically, the constitutive equations are:

$$\sigma_{\mathrm{R}}+\varepsilon E^2 = \lambda_{\mathrm{R}} \cdot \frac{\partial W(\lambda_{\mathrm{R}}, \lambda_{\theta})}{\partial \lambda_{\mathrm{R}}}, \tag{1}$$

$$\sigma_{\theta}+\varepsilon E^2 = \lambda_{\theta} \cdot \frac{\partial W(\lambda_{\mathrm{R}}, \lambda_{\theta})}{\partial \lambda_{\theta}}. \tag{2}$$

In Equations (1) and (2), σ_{R} and σ_{θ} are the radial and circumferential real normal stress in the polar coordinate system, respectively; ε and E are the permittivity and nominal electric field strength in the direction perpendicular to the DE plane, respectively; λ_{R} and λ_{θ} ($\lambda_{\mathrm{R}} = \varepsilon_{\mathrm{R}} + 1$; $\lambda_{\theta} = \varepsilon_{\theta} + 1$, in which ε_{R} and ε_{θ} represent the radial and circumferential nominal strain, respectively) represent the radial and circumferential stretch ratios relative to the reference configuration, respectively; $W(\lambda_{\mathrm{R}}, \lambda_{\theta})$ is the function of strain energy based on the Gent constitutive model:

$$W(\lambda_{\mathrm{R}}, \lambda_{\theta}) = -\frac{1}{2}\mu J_{\mathrm{m}} \cdot \ln\left(1 - \frac{\lambda_{\mathrm{R}}^{2} + \lambda_{\theta}^{2} + \lambda_{\mathrm{R}}^{-2}\lambda_{\theta}^{-2} - 3}{J_{\mathrm{m}}}\right). \tag{3}$$

In Equation (3), μ and J_{m} are the two material parameters in the Gent constitutive model.

The equilibrium equation of the substrates is

$$\frac{dS_R}{dR} + \frac{S_R - S_\theta}{R} = 0. \tag{4}$$

In Equations (4), S_R and S_θ are radial and circumferential nominal normal stress, respectively. R represents the distance from the origin of the polar coordinate system in the reference configuration. The equation of compatibility is:

$$\frac{\partial \lambda_R}{\partial R} = \frac{1}{R}(\lambda_R - \lambda_\theta). \tag{5}$$

Combining equations (1) ~ (5), we can obtain the following two ordinary differential equations (ODEs):

$$\frac{\partial \lambda_R}{\partial R} = [\frac{\partial^2 W}{\partial \lambda_R{}^2} - \varepsilon(\frac{\phi}{H})^2 \lambda_\theta{}^2]^{-1} \times$$

$$\left\{\frac{1}{R}(\lambda_R - \lambda_\theta)[2\varepsilon(\frac{\phi}{H})^2 \lambda_R \lambda_\theta] - \frac{1}{R}[\frac{\partial W}{\partial \lambda_R} - \frac{\partial W}{\partial \lambda_\theta} + \varepsilon(\frac{\phi}{H})^2 \lambda_R{}^2 \lambda_\theta - \varepsilon(\frac{\phi}{H})^2 \lambda_R \lambda_\theta{}^2]\right\}, \tag{6}$$

$$\frac{\partial \lambda_\theta}{\partial R} = \frac{1}{R}(\lambda_R - \lambda_\theta). \tag{7}$$

In Equations (7), ϕ and H are the potential of the electrodes along the direction perpendicular to the DE plane and the initial thickness (e.g., H = 1 mm) of the DE membrane before pre-stretching, respectively.

Other equations between important variables are listed below:

$$E = \frac{\phi}{H}, \tag{8}$$

$$S_R = \frac{\sigma_R}{\lambda_R}, \tag{9}$$

$$S_\theta = \frac{\sigma_\theta}{\lambda_\theta}, \tag{10}$$

The nonlinear governing equations were solved numerically by adopting a shooting method. We investigate the deformation of 'region C' firstly, as the boundary conditions are known. In this case, the potential ϕ was assigned to 'region B' ($\phi = 0$ in other regions) and $\lambda_{\mathrm{B-C}}$ was assumed a trial value, where $\lambda_{\mathrm{B-C}}$ represents the circumferential stretch ratio at radius b ($\lambda_{\mathrm{B-C}} = \lambda_{\theta}$ at $R = b$); 'A', 'B' and 'C' represent three different regions; and 'a', 'b' and 'c' are the radii of three circular edges in **Supplementary Fig. 4c**, available as Supplementary Data at NSR online. The circumferential stretch ratio at radius c is given by $\lambda_{\mathrm{pre}} = \lambda_{\theta}$ ($R = c$), which is prescribed according to the pre-stretching process. Then the shooting method were used to obtain λ_{R} at $R = b$ in 'region C', and S_{R} at $R = b$ can be obtained based on equation (1).

Then we investigate the deformation of 'region B', in which the computed boundary conditions (S_{R} at $R = b$ and $\lambda_{\mathrm{B-C}}$) in the first step were substituted into equation (1) to obtain the λ_{R} at $R = b$ in B region. Then $\lambda_{\mathrm{A-B}}$ (λ_{R} at $R = a$) and S_{R} at $R = a$ can be determined.

Lastly, we investigate the deformation of 'region A', in which λ_{R} equals to λ_{θ} due to the equal biaxial deformation in 'region A'. Finally, we need to verify if the boundary conditions ($\lambda_{\mathrm{A-B}}$ and S_{R} at $R = a$) satisfy equation (1). If the boundary conditions indeed satisfy equation (1) or the discrepancy is sufficiently small, then the $\lambda_{\mathrm{B-C}}$ assumed in the first step is regarded the correct solution. Otherwise, we need to return to the first step to update the value of $\lambda_{\mathrm{B-C}}$ and repeat the above three steps until the verification is satisfied.

Supplementary Movie 1: A reconfigurable 3D mesostructure based on the electrically-controlled DE substrate, with use of two different loading paths.

Supplementary Movie 2: A dome-like mesostructure actuated rapidly by a circular electrode.

Supplementary Movie 3: An insect-like reconfigurable mesostructure exhibiting a different gesture after the rapid electrical actuation.

Supplementary Movie 4: The demonstration of the tunable capacitor device to adjust the light intensity of LEDs.

Supplementary Figures

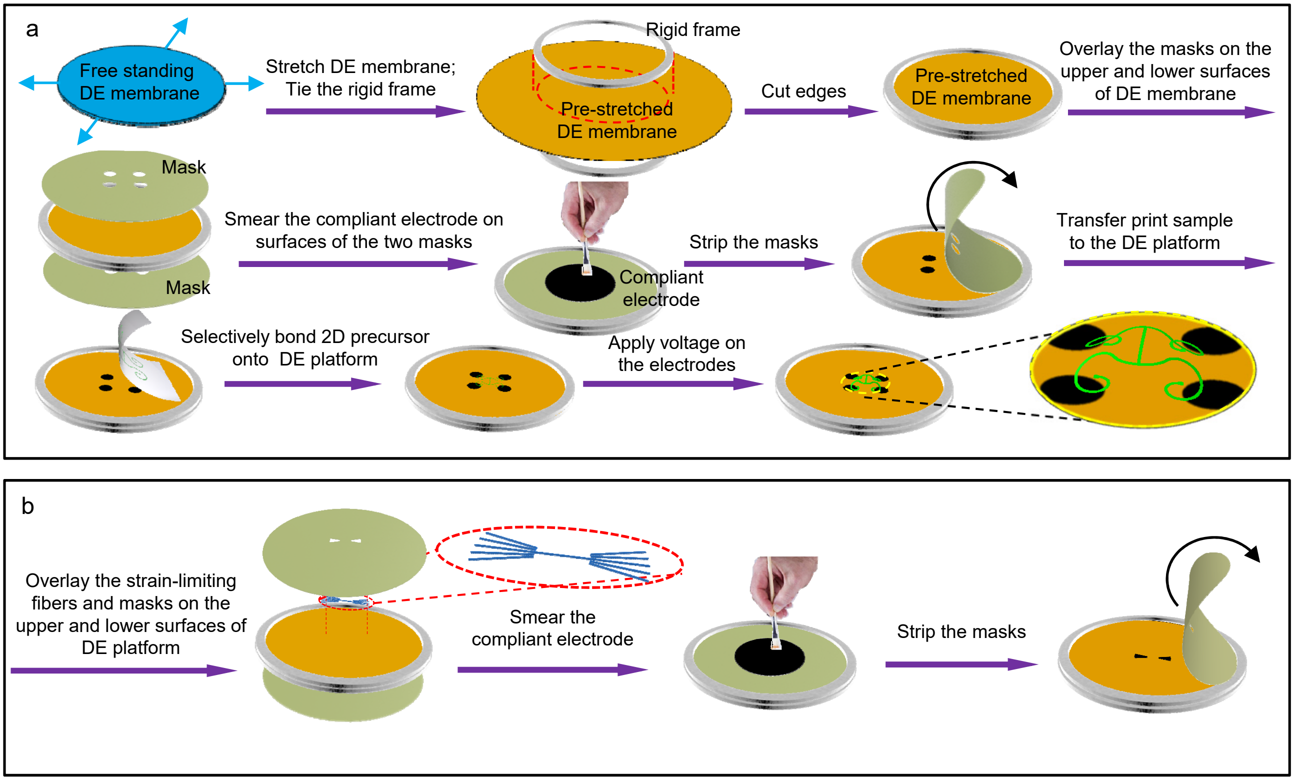

Supplementary Figure 1 | a, The manufacturing process for the assembly of 3D mesostrucutres based on electrically-controlled DE platforms. **b**, The manufacturing process of composite DE substrates consisting of strain-limiting fibers (see 'Methods' section for preparation of fibers, available as Supplementary Data at NSR online).

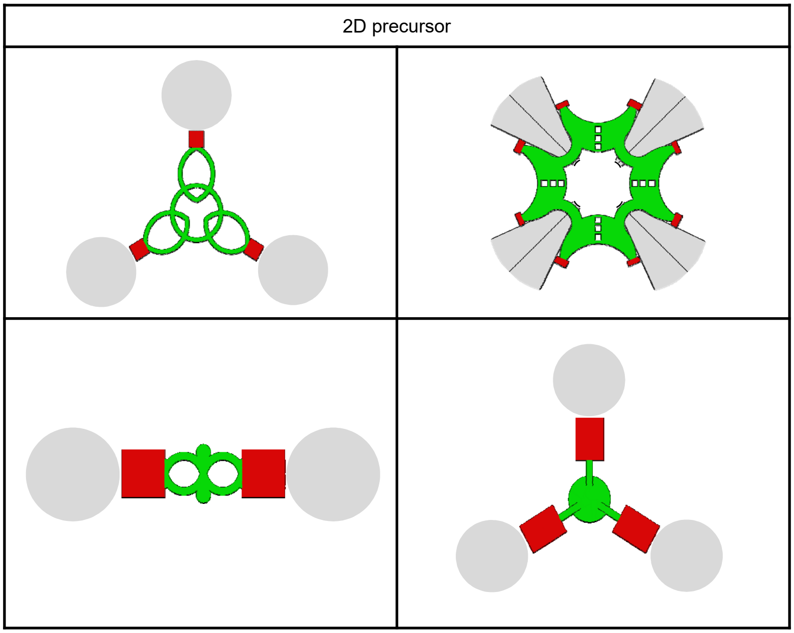

Supplementary Figure 2 | Schematic illustration for the designs of electrode layouts and 2D precursors in **Figure 1 c**, with electrodes and strain-limiting fibers marked in gray and black, respectively.

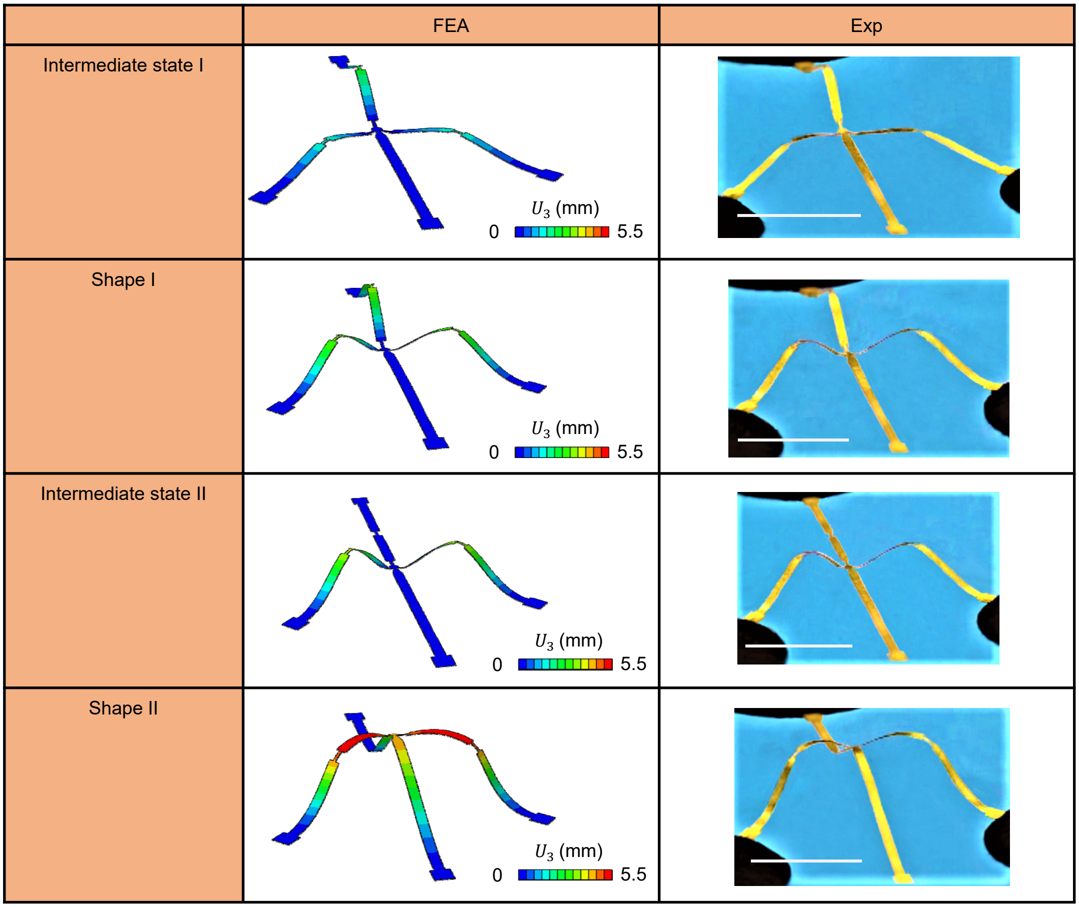

Supplementary Figure 3 | FEA results and optical images for the 3D reconfigurable mesostrucutre in **Figure 1 e**. Colors in FEA results represent the magnitude of the out-of-plane displacement of 3D mesostructures. Scale bars, 9 mm.

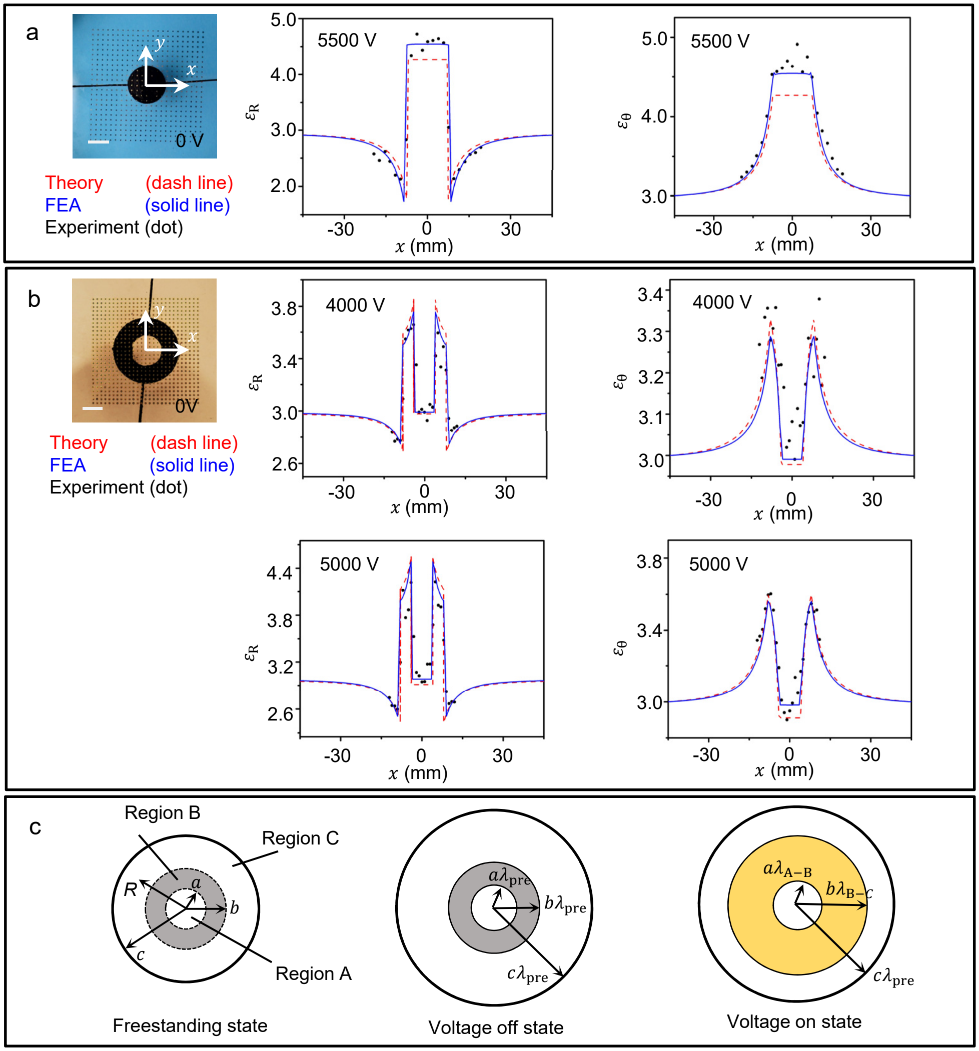

Supplementary Figure 4 | a, The FEA, experimental and theoretical results of the distributions of circumferential and radial nominal strain along x axis of the circular electrode at 5500 V. ε_R and ε_θ represent radial and circumferential nominal strain. **b**, Similar results for the annular electrode at two different levels of applied voltages (i.e., 4000/5000 V). **c**, Schematic illustration for the three states of DE substrates (i.e., 'freestanding state' without any pre-stretching, 'voltage off state' with pre-stretching and 'voltage on state' with pre-stretching at an applied voltage). Three regions ('A', 'B' and 'C') are illustrated, with 'a', 'b', 'c' representing the radius of three circular edges connecting each of the three regions, respectively. 'R' represents the distance from any point in the reference configuration (the 'freestanding state') to the center point of the annulus. $\lambda_{\mathrm{A-B}}$, $\lambda_{\mathrm{B-C}}$ and λ_{pre} represent λ_{R} at $R = a$, λ_θ at $R = b$ and λ_θ at $R = c$, respectively. Scale bars, 5 mm.

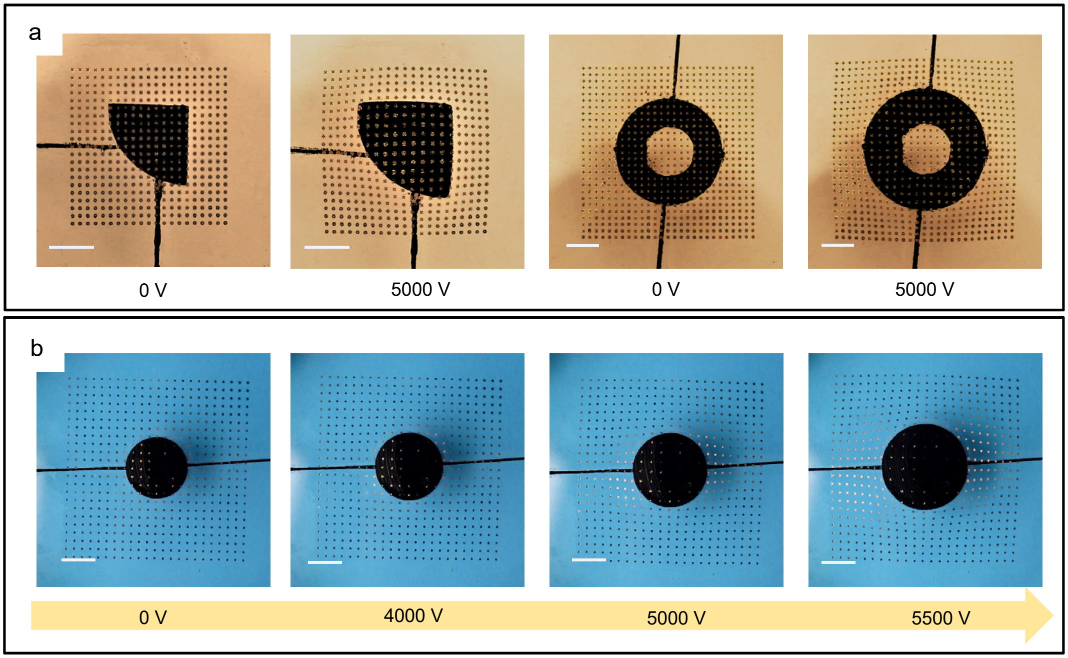

Supplementary Figure 5 | a, Optical images of the sector (without any strain-limiting fibers) and annular electrodes with deposited metal dots arrays at two different levels of applied voltages (0/5000 V). **b**, Similar results for the circular electrode at four different levels of applied voltages (0/4000/5000/5500 V). Scale bars, 5 mm.

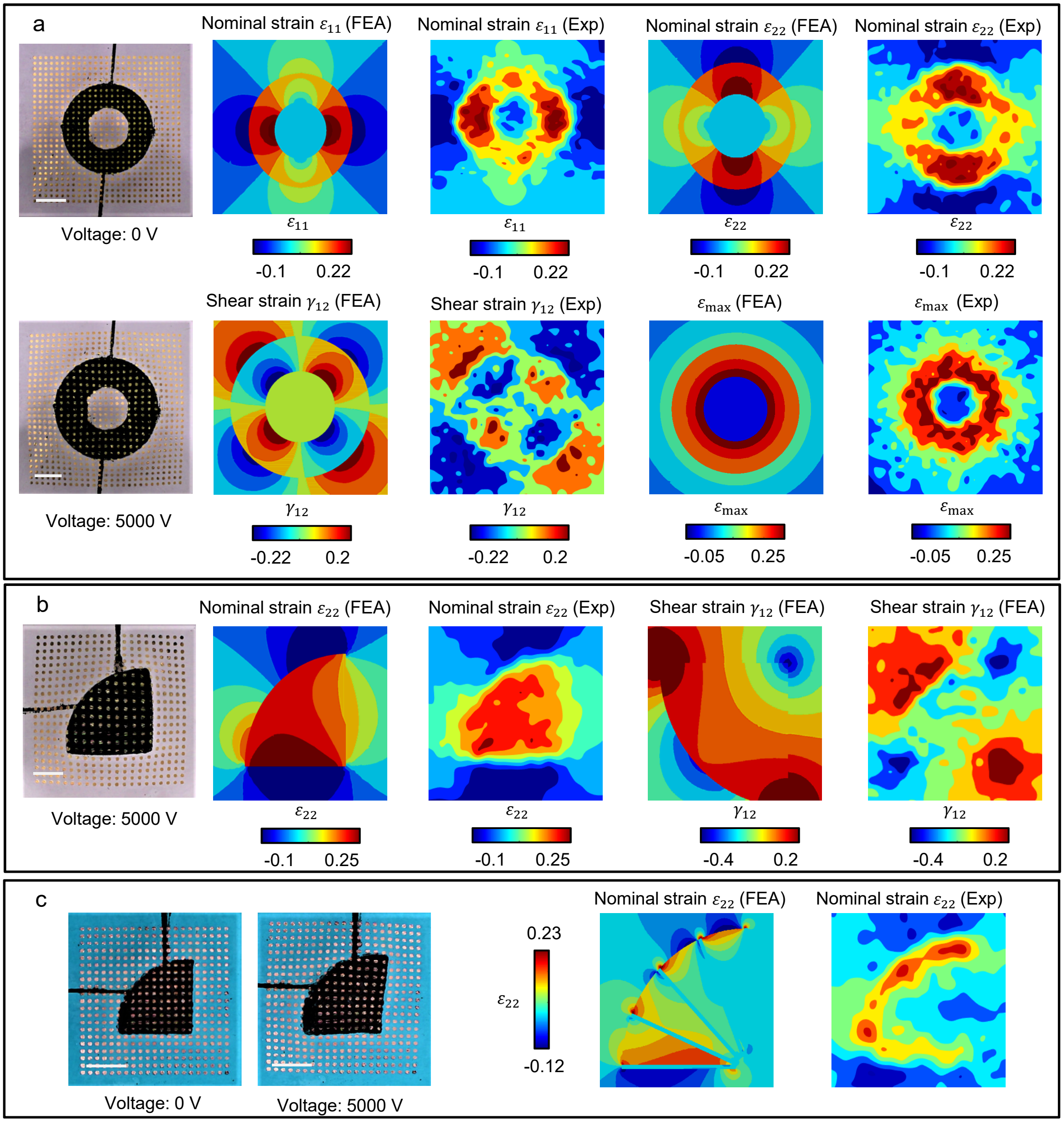

Supplementary Figure 6 | a, Optical images of the un-actuated (0 V) and actuated (5000 V) configurations of the DE substrate with an annular electrode and the distributions of strain components (ε_{11}, ε_{22}, γ_{12} and ε_{max}) determined from the experiment and FEA. **b**, Optical images of the actuated (5000 V) configuration of the DE substrate with an sector electrode, and the distributions of strain components (ε_{22} and γ_{12}) determined from the experiment and FEA. **c**, Optical images of the un-actuated (0 V) and actuated (5000 V) configurations of the DE substrate having sector electrode with strain-limiting fibers, and the distributions of strain component (ε_{22}) determined from the experiment and FEA. Scale bars, 5 mm.

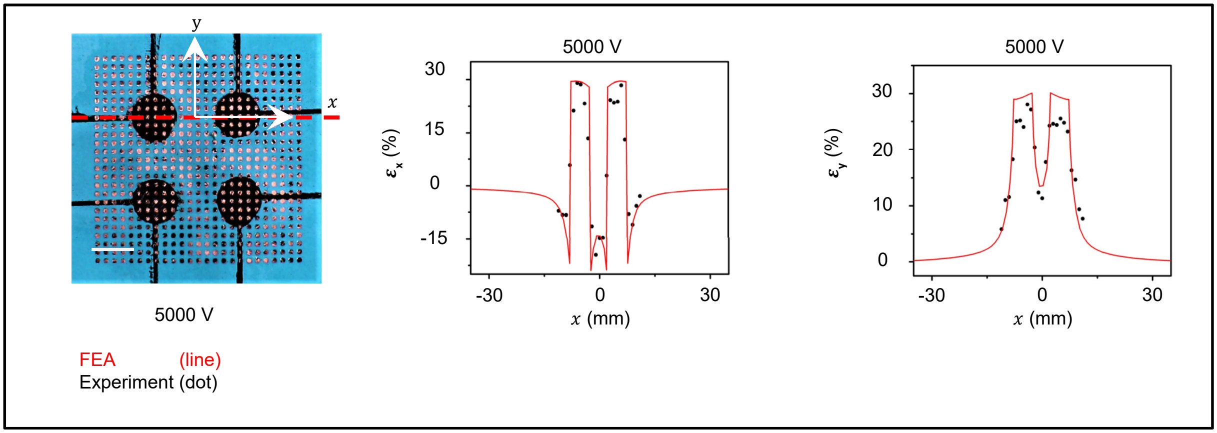

Supplementary Figure 7 | Optical image, as well as FEA and experimental results of the distributions of strain components (normal strain ε_x and ε_y in the x direction and y direction, respectively) for the 2×2 array of circular electrodes, along the line connecting the centers of the two electrodes (the red dash line in optical image) at 5000 V. Scale bars, 5 mm.

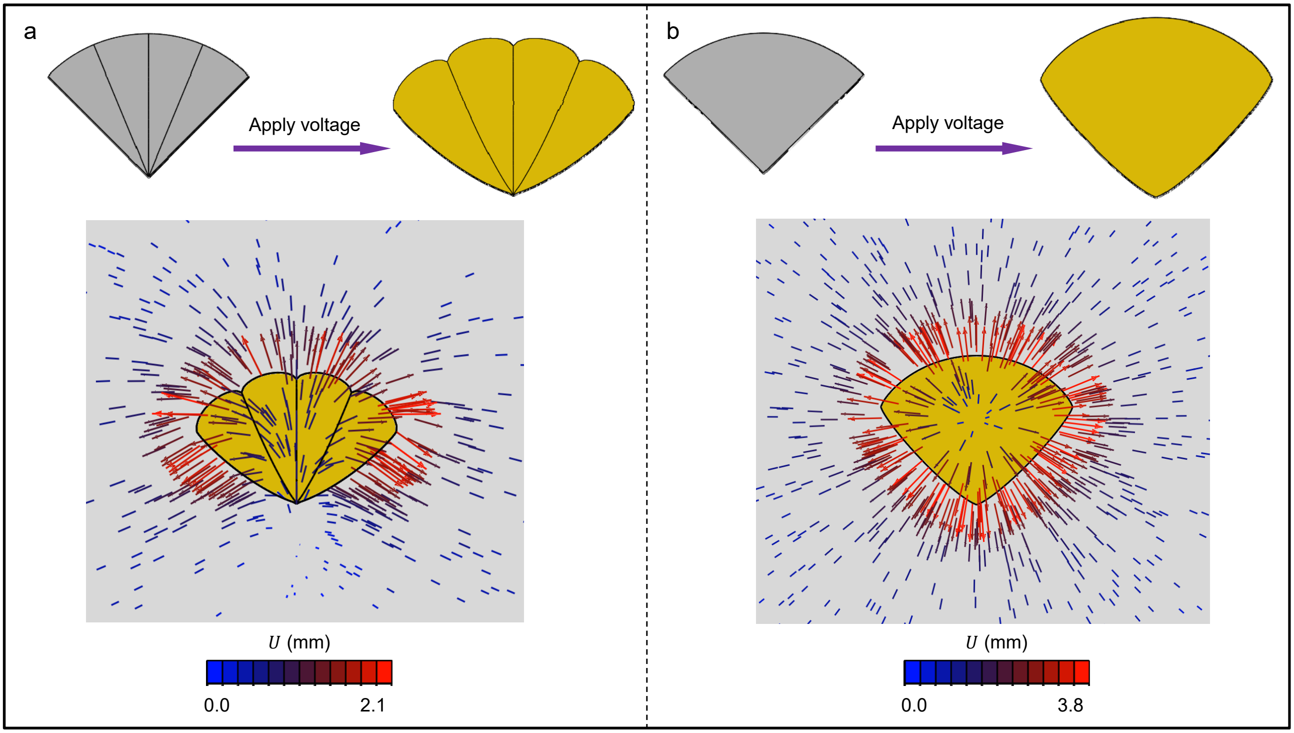

Supplementary Figure 8 | a, Result of FEA that illustrates the deformation and the distribution of displacement vectors of DE substrate having a sector electrode with strain-limiting fibers, at a level of applied voltage. **b**, Similar results for the sector electrode without any strain-limiting fibers.

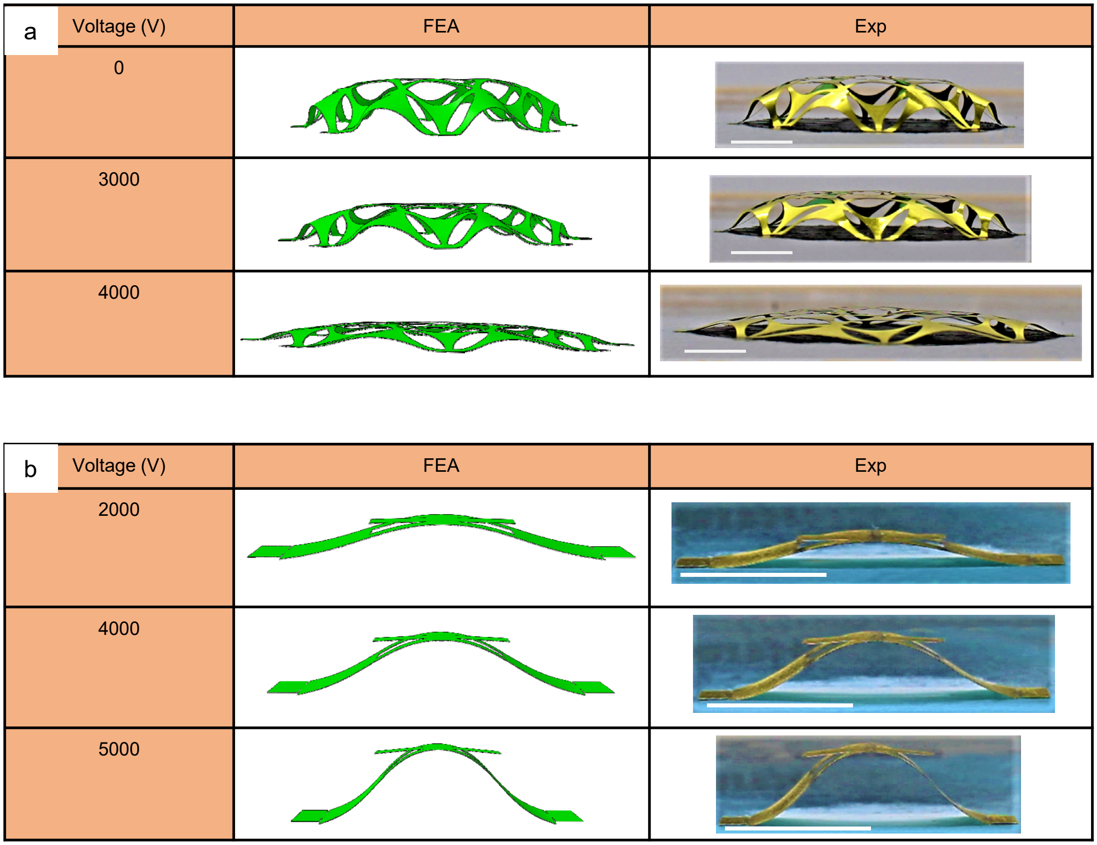

Supplementary Figure 9 | a, FEA and experimental images of the mesostructure in **Figure 3 a** at three different levels of applied voltages (0/3000/4000 V). **b**, Similar results for the mesostructure in **Figure 3 b** at three different levels of applied voltages (2000/4000/5000 V). Scale bars, 10 mm.

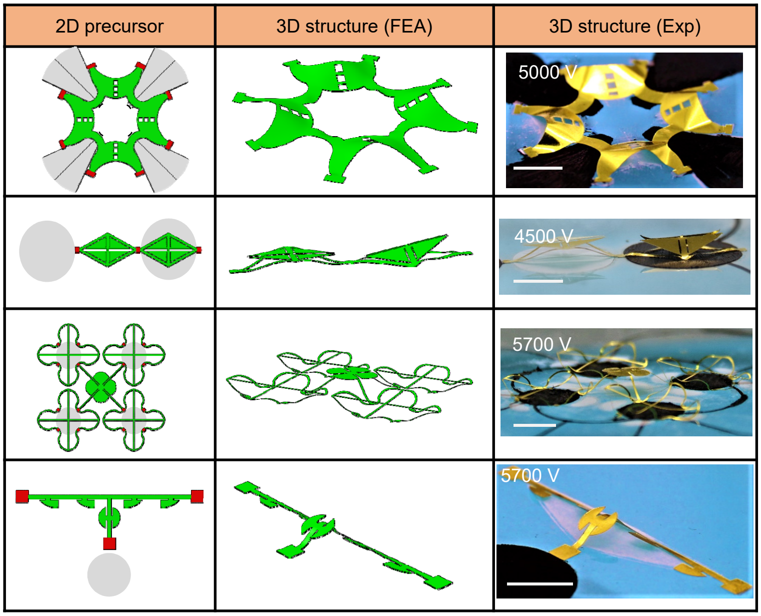

Supplementary Figure 10 | Experimental and FEA results of four other mesostructures achieved by electrically-controlled 3D assembly. The electrodes, fibers and bonding sites are marked by grey, black and red in the schematics of 2D precursor, which are made of Al/PET (2.5 μm/30 μm). Scale bars, 10 mm.

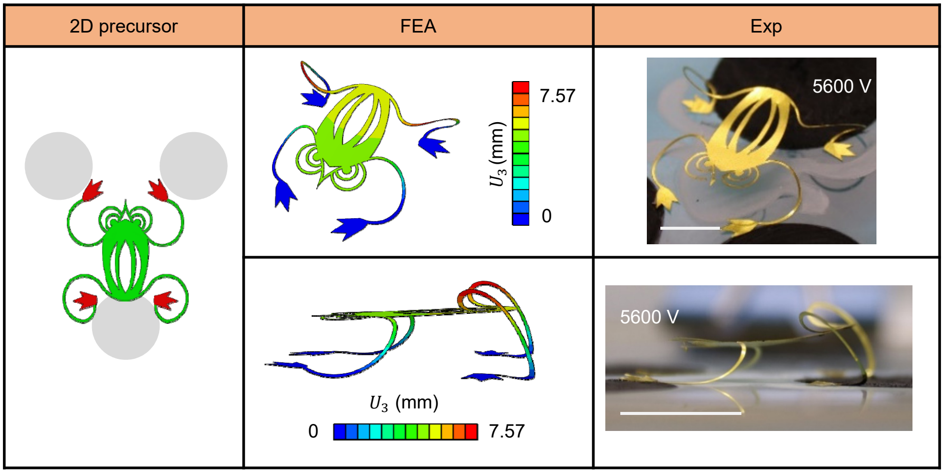

Supplementary Figure 11 | FEA and experimental results of the frog-like mesostructure in **Figure 3 d**. Colors in the FEA results represent the magnitude of out-of-plane displacements of the 3D mesostructure. Scale bars, 8 mm.

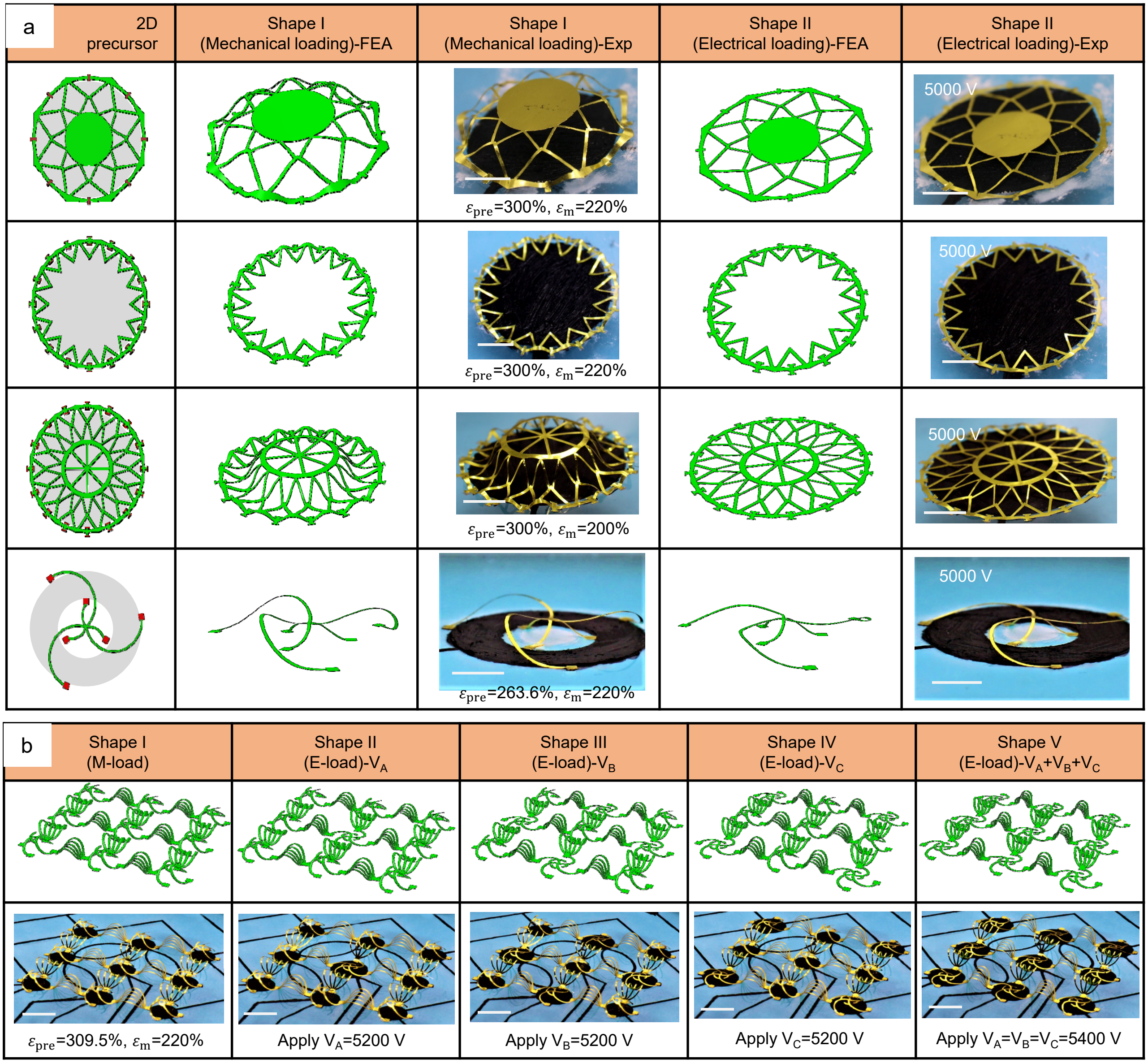

Supplementary Figure 12 | FEA and experimental results of some 3D reconfigurable mesostructures. **a**, Experimental and FEA results of four other reconfigurable mesostructures with coupled mechanical and electrical loadings. **b**, FEA and optical images of the 3D reconfigurable network mesostructure in **Figure 3 i**. 'M-load' and 'E-load' represent 'Mechanical loading' and 'Electrical loading', respectively. Scale bars, 10 mm.

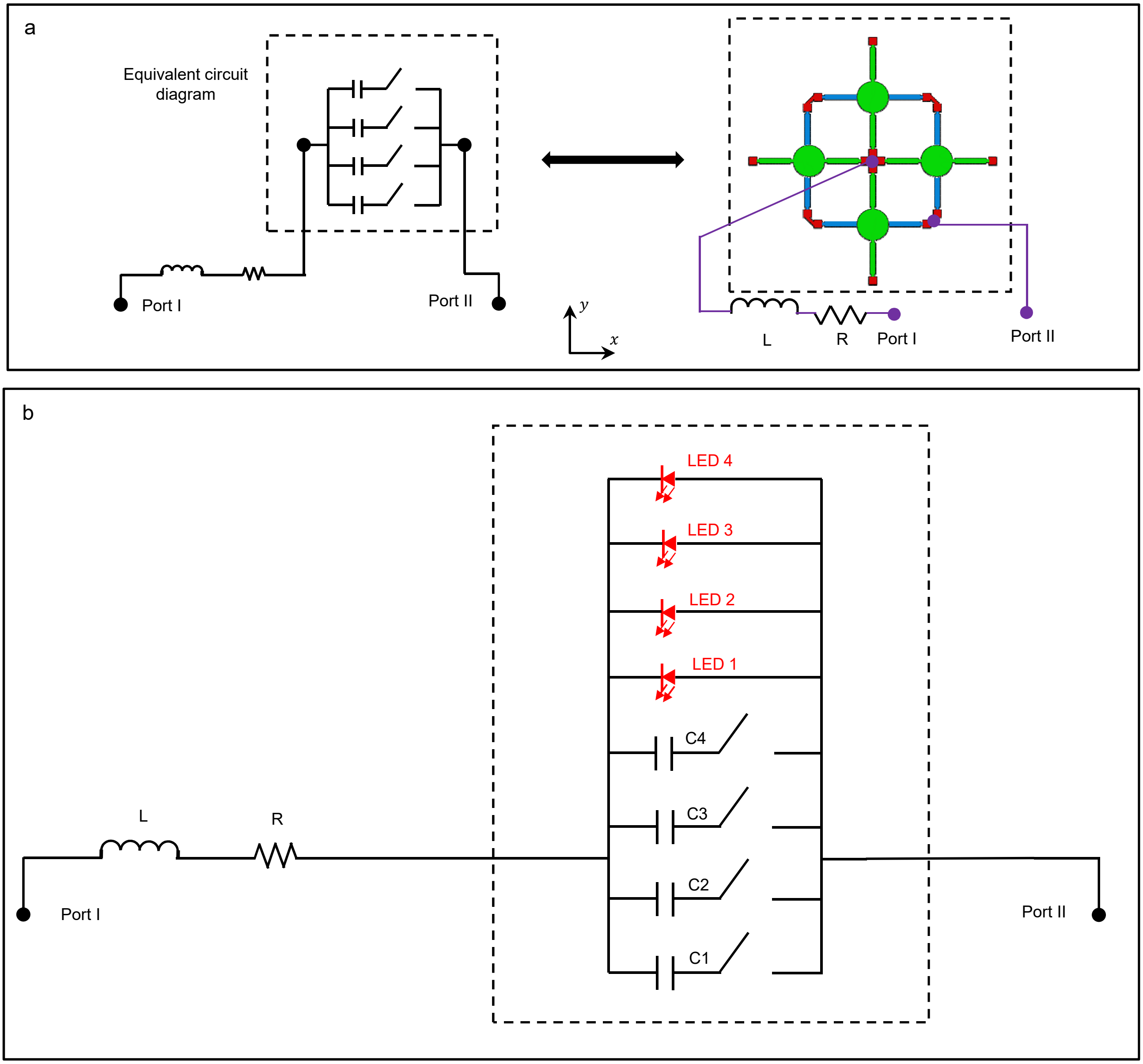

Supplementary Figure 13 | The circuit diagram in the planar state and the equivalent circuit of the functional device in **Figure 4 b**. **a**, The equivalent LC-RF circuit with four capacitors connected in parallel (in the dashed box; the four capacitors can be switched on and off thanks to the reconfiguration of the 3D device). Port I and port II are the two circuit ports. **b**, The equivalent circuit of the tunable capacitor device with LEDs in **Figure 4 e**, in which four LEDs are connected in parallel to the four capacitors.

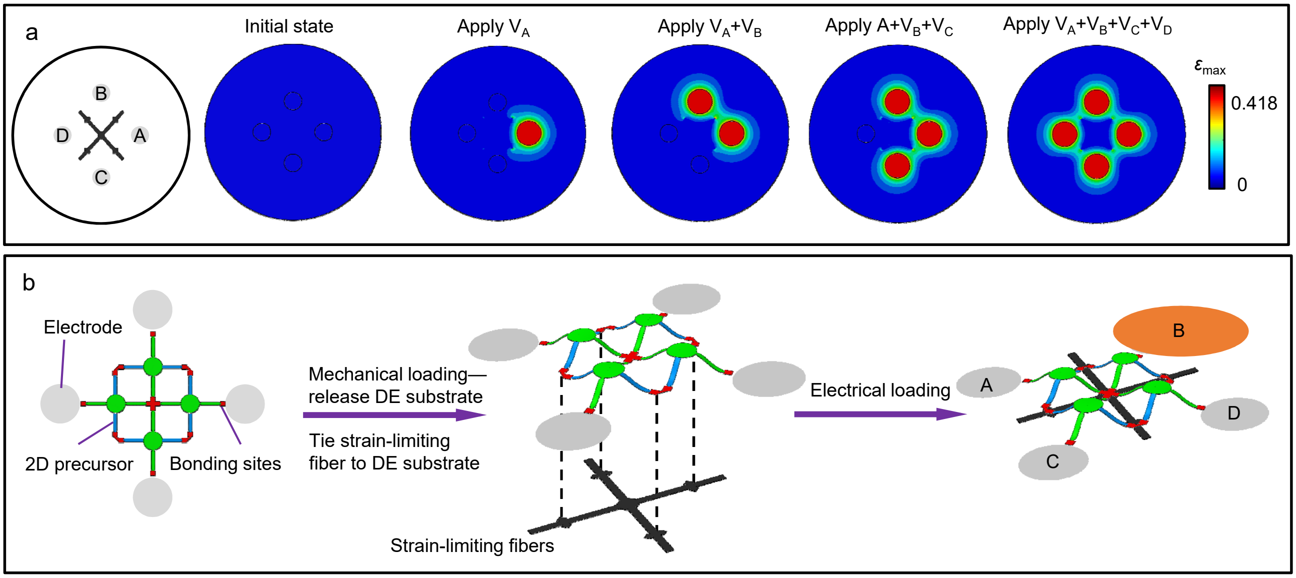

Supplementary Figure 14 | FEA results of 3D reconfigurable device, as well as the distributions of strain of DE substrate with the designed electrode layout in the LC-RF circuit. **a**, The distributions of strain of DE substrate of the functional device in **Figure 4**, under four different types of electrical loadings (‘V_A’, ‘V_A+V_B’, ‘V_A+V_B+V_C’ and ‘V_A+V_B+V_C+V_D’ applied at 5200 V). Colors in FEA results represent the magnitude of in-plane maximum principal strain (ε_{max}). **b**, The manufacturing process of the fabrication of the 3D reconfigurable device derived from FEA.

Reference

1 Koh, SJA, Li, T, Zhou, J*, et al*. Mechanisms of large actuation strain in dielectric elastomers. *J Polym Sci, Part B: Polym Phys* 2011; **49**: 504-15.

2 Suo, Z. Theory of dielectric elastomers. Acta Mechanica Solida Sinica 2010; **23**: 549-78.
